# 2769. Treatment of Hypermutator *Pseudomonas aeruginosa* Induces Multidrug Resistance but Can Be Targeted by Combination Therapy

**DOI:** 10.1093/ofid/ofad500.2380

**Published:** 2023-11-27

**Authors:** Kalen M Hall, William C Wimley, Christine M Bojanowski, Zachary F Pursell, Lisa Morici

**Affiliations:** Tulane University School of Medicine, New Orleans, Louisiana; Tulane University School of Medicine, New Orleans, Louisiana; Tulane University School of Medicine, New Orleans, Louisiana; Tulane University School of Medicine, New Orleans, Louisiana; Tulane University School of Medicine, New Orleans, Louisiana

## Abstract

**Background:**

Up to 60% of *Pseudomonas aeruginosa* (Pa) strains isolated from human chronic lung infections lack an active DNA mismatch repair (MMR) system, resulting in a hypermutator phenotype. Hypermutator Pa isolates are associated with enhanced multidrug resistance and treatment failure, and treatments targeting hypermutators are not available. Although hypermutation has been shown to catalyze drug resistance acquisition, it is unclear how treatment of hypermutator Pa influences the development of multidrug resistance.

**Methods:**

Here, we performed *in vitro* adaptive evolution of hypermutator Pa under different antibiotic conditions to assess if treatment of hypermutator Pa induces multidrug resistance acquisition via repeated selection for specific resistance-conferring mutations. We serially exposed hypermutator Pa and wild type Pa to four different antibiotics representing three drug classes, and subsequently tested evolved lineages against a panel of six non-treatment antibiotics from varying classes.

**Results:**

We observed that hypermutator Pa acquired levels of resistance above clinical breakpoints (defined by Clinical and Laboratory Standards Institute) to all antibiotics tested faster than wild type Pa. Antibiotic treatment of hypermutator Pa selectively induced multidrug resistance acquisition but was irrespective of drug class. Using whole genome sequencing of resistant lineages, we found that treatment-induced multidrug resistance acquisition was determined by shared mechanisms of resistance rather than shared cell target. We subsequently tested combination therapies requiring independent and exclusive mechanisms of resistance and found that combined aztreonam/colistin effectively eliminated multidrug resistance acquisition in hypermutator Pa. Currently, we are testing this combination therapy on 14 Pa isolates (with 4 hypermutator and 10 wild type) from cystic fibrosis patients.

**Conclusion:**

Our results shed light on how hypermutator Pa may acquire multidrug resistance within the host and suggest that combination therapies that require independent mechanisms of resistance could potentially reduce emergence of multidrug resistance in hypermutator Pa, providing a viable therapeutic option.

**Disclosures:**

**Kalen M. Hall, B.S., B.A.**, Informuta: 1|Informuta: Ownership Interest|Informuta: Stocks/Bonds

